# The transmission dynamics of *Campylobacter jejuni* among broilers in semi-commercial farms in Jordan

**DOI:** 10.1017/S0950268818003308

**Published:** 2019-03-08

**Authors:** M. I. Neves, I. Malkawi, M. Walker, A. Alaboudi, E. Abu-Basha, D. P. Blake, J. Guitian, M. Crotta

**Affiliations:** 1 Veterinary Epidemiology, Economics and Public Health Group, The Royal Veterinary College, Hawkshead Lane, North Mymms, AL9 7TA, Hatfield, UK; 2 Faculty of Veterinary Medicine, Jordan University of Science and Technology, Irbid, Jordan

**Keywords:** Bayesian analysis, broiler chicken, campylobacter, Jordan

## Abstract

Campylobacter is the leading cause of foodborne bacterial gastroenteritis in humans worldwide, often associated with the consumption of undercooked poultry. In Jordan, the majority of broiler chicken production occurs in semi-commercial farms, where poor housing conditions and low bio-security are likely to promote campylobacter colonisation. While several studies provided estimates of the key parameters describing the within-flock transmission dynamics of campylobacter in typical high-income countries settings, these data are not available for Jordan and Middle-East in general. A Bayesian model framework was applied to a longitudinal dataset on *Campylobacter jejuni* infection in a Jordan flock to quantify the transmission rate of *C. jejuni* in broilers within the farm, the day when the flock first became infected, and the within-flock prevalence (WFP) at clearance. Infection with *C. jejuni* is most likely to have occurred during the first 8 days of the production cycle, followed by a transmission rate value of 0.13 new infections caused by one infected bird/day (95% CI 0.11–0.17), and a WFP at clearance of 34% (95% CI 0.24–0.47). Our results differ from published studies conducted in intensive poultry production systems in high-income countries but are well aligned with the expectations obtained by means of structured questionnaires submitted to academics with expertise on campylobacter in Jordan. This study provides for the first time the most likely estimates and credible intervals of key epidemiological parameters driving the dynamics of *C. jejuni* infection in broiler production systems commonly found in Jordan and the Middle-East and could be used to inform Quantitative Microbial Risk Assessment models aimed to assess the risk of human exposure/infection to campylobacter through consumption of poultry meat.

## Introduction

Campylobacter is considered to be the leading cause of bacterial gastroenteritis in humans worldwide. In high-income countries, infection is mainly caused by the consumption of contaminated undercooked chicken meat, ready-to-eat products or cross-contamination from raw chicken to other foods. Recently, the European Food Safety Authority (EFSA) estimated that the consumption of chicken meat may account for 20–30% of the total cases of campylobacteriosis in the EU [1], and for this reason, prevention of human infection with campylobacter focuses predominantly on reducing its presence in broiler chicken meat [1].

In 2011, EFSA's scientific opinion observed that the prevalence of campylobacter in broiler flocks and the risk to public health are in linear relationship. Although this assumed relationship represented a simplification, a quantitative risk assessment based on data from European countries estimated that a reduction of 3 log_10_ colony forming units (CFU) in the numbers of campylobacter in the chicken's intestine at slaughter could reduce the risk of human infection by at least 90% [2].

A few studies in low- and middle-income countries (LMICs) have also shown that poultry meat may be an important source of campylobacteriosis [3–5]. Jordan is an Arab Kingdom in the Middle East, with semi-arid characteristics and a population of 9.9 million people in 2017 [6]. Poultry production is a major contributor to food security in Jordan, with a per capita broiler meat production of nearly 22 kg [7]. The majority of the production occurs in semi-commercial farms, which produce up to around 5000 birds per production cycle (lasting typically from 30 to 35 days). The poor housing conditions, low biosecurity and lack of skilled professionals and surveillance programmes are thought to contribute to the colonisation of broilers by campylobacter in these farms [8]. Poultry production is particularly important for the development of Jordan, since agricultural production is not currently sufficient to support the increasing needs derived from the continual arrival of Syrian refugees. Sufficient and safe poultry meat production is therefore essential to ensure food security and narrow the protein supply gap; however, national regulations have not yet been established to control campylobacter in live and dressed chicken in Jordan [8].

Campylobacter infection in poultry farms is a multifactorial event with the main risk factors related to the environment, the management and the birds themselves [2]. A dose as low as 40 CFU has been observed as sufficient to infect a chicken in experimental conditions [9]; however, it should be considered that this dose and the kinetics of colonisation in chicken broiler may be strain and breed dependent [10, 11]. After infection, the microorganism usually reaches high concentrations in caeca very quickly and is shed with faeces [12, 13]. Cases of self-limiting infections have been occasionally reported [10], but considering chicken broiler reared in intensive systems and the length of the production cycle (usually <50 days), it is generally accepted that once a bird is infected, the infection persists until clearance [10]. Since chickens are coprophagic, faecal shedding is an important mechanism for the within-herd transmission of infection. While several longitudinal studies have shown how campylobacter spreads very rapidly among broilers [14–17], there is still considerable uncertainty in key factors modulating the transmission dynamics of campylobacter at farm level, particularly: (i) the time when birds first become infected and (ii) the effects of environmental/managerial factors on the rate of transmission. If these parameters are better understood, interventions could be implemented more efficiently by targeting them to periods of greatest risk [18] and reduce the farm-level prevalence of campylobacter at the time of slaughter [19, 20].

In high-income countries, different studies have estimated these parameters for broiler farms with commercial/intensive production systems operating under strict biosecurity measures and highly qualified professionals [21, 22]. To the extent of our knowledge, these parameters have never been explored in the semi-commercial settings of the Middle East and LMICs in general. Therefore, the objectives of this study were to estimate: (i) the within-flock rate of transmission of *Campylobacter jejuni*, (ii) the day when the flock first became infected, and (iii) the within-flock prevalence (WFP) at clearance in a semi-commercial broiler farm in Jordan, which has a production system that is commonly found in many low- and middle-income settings across the world.

## Methods

### Field study design and data collection

A longitudinal study was carried out in one semi-commercial broiler farm in Irbid (Jordan), between May and June 2017. Semi-commercial broiler production systems in Jordan are characterised by farms having up to 5000 birds per cycle, a production cycle of around 40 days, housing of concrete buildings with metallic roofs, natural lighting and ventilation, manual feeders and automatic water supply, management done by the owner and low-biosecurity measures [7]. The studied flock consisted of 5000 birds obtained from a local hatchery, kept at a density of around 10 birds/m^2^, and a broiler production cycle of 35 days. Sampling of chicks was conducted at fixed time points during the cycle (starting at day 5). At each sampling age, 50 chicks were randomly chosen and removed from the group. One cloacal swab was collected from each animal by the local veterinarian, and after sampling, the chicks were placed back into the flock. Additionally, one boot sample (a sock was put on the base of a pair of boots and used to collect faecal material while the veterinarian walked around the flock) was collected on the same sampling day. This approach was used previously to test for the presence of *C. jejuni* in the environment [23].

The fixed time points for collection of samples were on days 5, 10, 13, 15, 17, 20, 25 and 35. Previous studies reported that 1–2 weeks old chickens may be less susceptible to colonisation [24, 25] with the presence of maternal antibodies in young chickens suggested as an explanation for this time lag [26]. Considering this and the rapid within-flock spread of campylobacter once the first bird becomes infected, this sampling plan was designed to increase the amount of data gathered around the time of fast spread within the flock, as this is the period when change occurs more rapidly and therefore when data are more informative for our estimates. The relationship between sampling lag and uncertainty in estimates is shown in the paper by Goddard *et al*. and this study recommended that sampling should occur at intervals of <1 week [21].

### Isolation and identification of *C. jejuni*

A total of 408 samples (400 cloacal swabs and eight boot samples) were analysed in the Food Safety Research Laboratory at JUST (Jordan University of Science and Technology). Detection/identification of *C. jejuni* colonies, DNA extraction and polymerase chain reaction (PCR) were performed following the procedures reported in the ISO 10272-1:2006 [27] and Osaili *et al*. [8]. Briefly, cloacal and boot samples were collected by a veterinarian using sterile cotton swabs, and added to transport media (alkaline peptone water with sodium thioglycollate and L-cysteine) [23]. Samples were subsequently transported aseptically in a cool box with ice to the laboratory. Campylobacter was detected as described in ISO FDIS 10272-1 (2006), by enrichment in Bolton Broth (Oxoid) at 37 °C for 4 h and 42 °C for 44 h under microaerobic conditions created using the OxoidCampyGen system. Subsequently, samples were streaked on Preston agar selective media and plates were incubated at 41.5 °C for 48 h in a microaerophilic atmosphere. After incubation, plates were inspected for the presence of colonies with typical campylobacter morphology. Suspected campylobacter colonies were streaked at least twice into Colombia blood agar (Oxoid) and incubated at 42 °C for 48 h. The same plates were used for further characterisation [27].

#### Identification of *C. jejuni*

Confirmation of campylobacter spp. identity was achieved by colony morphology, hanging drop motility, catalase test, oxidase test and dry spot agglutination test [27]. Hippurate hydrolysis test was conducted to identify *C. jejuni* [27].

#### DNA extraction and PCR

Total genomic DNA was extracted by suspending individual colonies in 300 µl nuclease-free water and heating in a dry block for 10 min at 100 °C. The samples were then cooled immediately in an ice bath for 5–10 min and centrifuged at ~10 000 ***g*** for 5 min. The supernatants were used as DNA templates for PCR. Primers specific for *C. jejuni* were selected according to a previous study by Nayak *et al*. [28] targeting the nucleotide sequence of a putative oxidoreductase subunit annotated within the *C. jejuni* genome. The primers used to amplify the DNA segment were F5′-CAA ATA AAG TTA GAG GTA GAA TGT-3′ and R5′-GGA TAA GCA CTA GCT AGC TGA T-3′ (synthesised by Alpha DNA). The reaction mixture consisted of 12.5 µl of Promega green master mix (Promega, Madison, Wisconsin, USA), 1 µl of each primer, 1 µl DNA and 9.5 µl nuclease-free water. PCR amplification was performed on a Veritithermo-cycler (Applied Biosystems, Waltham, Massachusetts, USA) as follows: denaturation at 94 °C for 4 min, 33 cycles with denaturation at 94 °C for 1 min, annealing at 52 °C for 1 min and extension at 72 °C for 1 min, and the final extension at 72 °C for 5 min. The final PCR amplicons were examined by agarose gel electrophoresis, observed through a UV transilluminator and photographed with a GelDoc 2000 documentation system (Bio-Rad, Hercules, California, USA) [27].

### Estimating the transmission rate of campylobacter using a Bayesian approach

#### Modelling WFP

The WFP measures the expected number of birds infected with campylobacter within a positive flock [29]. It is generally accepted that after campylobacter infection, broilers shed the bacteria for the rest of their lives [30]. Thus, this model considers that the spread of campylobacter in a broiler flock can be represented by a susceptible-infected (SI) model. Changes in the number of susceptible *S*(*t*) and infected *I*(*t*)birds over time are described by
1
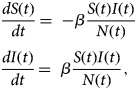

where *N*(*t*) = *S*(*t*) *+* *I*(*t*). This model assumes that, at all times, birds remain exclusively either susceptible or infected. Mortality of birds is not considered, since campylobacter primarily appears to act as a commensal organism in chickens [13]. Once a bird is infected, it cannot become susceptible again, since infection is generally thought to persist during the entire life span of a broiler [10]. The term *βS*(*t*)*I*(*t*)/*N*(*t*) defines the incidence of new infections per day. Substituting *S*(*t*) = *N*(*t*) − *I*(*t*) allows Eq. (1) to be reduced to a single equation and by further replacing *p*(*t*) = *I*(*t*)/*N*(*t*), the familiar expression for logistic growth is obtained from the analytical solution of the model
2
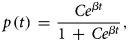

where,
3
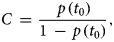

and *p*(*t*_0_) is the proportion of the flock initially positive for campylobacter. It is usually assumed that infection within a flock starts with just one bird. To allow the model to be more flexible and depart from this common assumption, we assumed that the proportion of birds at the start of infection within the flock, i.e. *p*(*t*_0_), is described by a uniform distribution which ranges from just one bird to 100 birds, assuming a flock size of 5000 birds. Equations (2) and (3) are re-parameterised [21], to facilitate (Bayesian) logistic regression such that
4
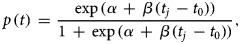

where,
5
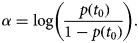



Here the logit of *p*(*t*) becomes a linear function with intercept *α* and gradient *β*, and *t* has been replaced by (*t*_*j*_ − *t*_0_) to describe the time passed since the flock first became infected until the sampling time, *t*_*j*_.

#### Defining prior distributions

A vague prior was assigned to the transmission rate parameter (the per capita contact rate per day) described by a *γ* distribution with shape parameter *k* = 0.001 and scale parameter *θ* = 0.001. Previous studies have suggested that infection with campylobacter in broilers can only be detected after the first 2–3 weeks of the cycle [18, 22]; however, for the purpose of this study, we decided to consider any day as a possible day of first infection. To this end, a vague prior described by a uniform distribution ranging between 0 and 35 days was selected for the day of first infection. Finally, a vague prior was assigned to the number of infected birds at the day of first infection, described by a uniform distribution ranging from 1 to 100 infected birds in a total of 5000 birds (Table 1).

**Table 1.** Description of the prior distributions included in the model for each parameter

Model parameter	Description	Prior value	Source
*t*_0_	Time when the flock became infected	Uniform (0, 35)	N/A
*Β*	Transmission rate parameter	*γ* (0.001, 0.001)	N/A
*p*(*t*_0_)	Proportion of infected birds at *t*_0_	Uniform (0.0002, 0.002)	N/A

#### Statistical inference

We estimated parameters *β* (transmission rate) and *t*_0_ (the time of first infection) by fitting the logistic regression model described by Eqs (4) and (5) to prevalence data from a broiler farm with 5000 birds using Bayesian Markov Chain Monte Carlo (MCMC) techniques implemented in JAGS and R [31, 32]. The model was fitted using three different starting values for each parameter to verify convergence on the posterior distribution [33]. The model was run for an initial ‘burn in’ period of 10 000 iterations and was further updated for 100 000 iterations to obtain an adequate approximation of the posterior distribution.

### Questionnaire

In parallel to the field study, a questionnaire aimed to obtain informative opinions on the expected WFP of campylobacter in a typical semi-commercial farm in Irbid was developed. The questionnaire was completed by two academics with expertise on campylobacter in Jordan from JUST University. The intent of the questionnaire was not to obtain accurate additional data, but to permit comparison of the answers from the two informed opinions with the real settings results, and to evaluate whether the perceptions of the experts on the prevalence of campylobacter in semi-commercial farms in Jordan were comparable to the results of the field study. Interviewees had to complete the questionnaire with estimates of the prevalence that would be expected if a longitudinal study was conducted in a semi-commercial broiler farm in Irbid and 50 samples were taken at each sampling point (mimicking the design of our sampling scheme). The characteristics of the farm, as well as the sampling points considered in this questionnaire, were similar to the ones included in the longitudinal study. The experts were not aware of the results obtained from our field study when they completed the questionnaire.

## Results

### Field study and data collection

Results of the longitudinal sampling conducted in one flock in the North of Jordan between May and June 2017 are reported in Table 2. The broiler flock became infected with *C. jejuni* during the production cycle, and it was first detected in samples taken when the chickens were 15 days old.

**Table 2.** Results obtained from the longitudinal study

Age of birds (days)	Number positive samples (out of 50)	Boot sample
5	0	−
10	0	−
13	0	−
15	4	+
17	4	+
20	5	+
25	7	+
35	15	+

### Posterior estimates

**Fig. 1.**
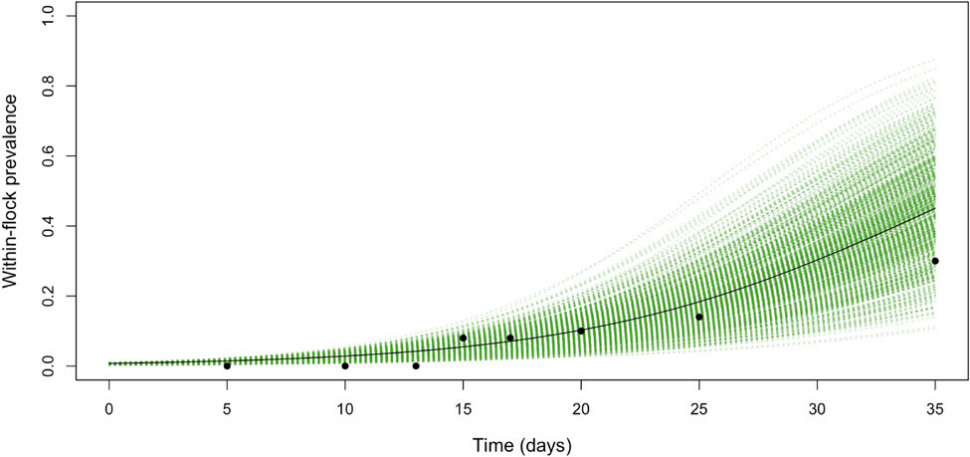
The within-flock prevalence dynamics for a 5000-bird flock in Jordan with a density of birds of around 10 birds/m^2^, obtained by fitting the model (including 95% credible intervals) to the longitudinal data points.

Table 3 shows the parameter posteriors estimated by fitting the WFP model to the collected data. The median estimate of the transmission coefficient, *β*, was 0.13 with 95% CI between 0.11 and 0.17 (Table 3). The median of the estimated posterior distribution of *t*_0_ was 2.71, with 95% CI between 0.11 and 8.29 days, suggesting that the flock became infected during the first 8 days of the production cycle, and that *C. jejuni* was introduced in the farm approximately 12 days before its detection. The median of the estimated posterior distribution of the prevalence within the flock on the last day of the cycle was 0.34, with 95% CI between 0.24 and 0.47. Raw longitudinal data points and fitted transmission model (including 95% credible intervals) are reported in Figure 1.

**Table 3.** Summary of the estimated posterior distributions obtained from fitting the within-flock transmission model to the longitudinal data on *C. jejuni* prevalence collected from a broiler farm in Jordan

Parameter	Description	Posterior estimates: median (2.5%, 97.5%)
*t*_0_	Day when the flock first became infected	2.71 (0.11, 8.29)
*Β*	Transmission rate (number of new infections per infected bird/day)	0.13 (0.11, 0.17)
Time of detection−time of first infection (*t*_0_)	Time until *C. jejuni* was detected after initial infection	12.29 days
WFP day 35	Prevalence of *C. jejuni* on day 35	0.34 (0.24, 0.47)

### Questionnaire

The final WFP of *C. jejuni* on day 35 was predicted by both experts to be 0.30 (15/50), well aligned with results of the field study (Table 4).

**Table 4.** Results from the questionnaire on the number of positive samples (out of 50) for campylobacter in a flock of 5000 birds expected by two academics with expertise on campylobacter

Age of birds	5	10	15	20	25	35
Expert 1	0^a^	0	2	4	10	15
Expert 2	0	0	0	3	10	15

a Values shown are the number of positive samples out of 50.

## Discussion

In this study, we have estimated the rate of transmission of *C. jejuni* among broiler chickens within a semi-commercial farm, the day when the flock first became infected with *C. jejuni*, and the WFP at clearance. These parameters, never explored before in Jordan and in semi-commercial broiler farms in the Middle East and LMICs in general, were estimated by fitting a simple SI transmission model in a Bayesian framework to longitudinal prevalence data.

The prevalence estimate obtained from the field study and the model are lower than those reported in the other studies previously mentioned, which have estimated it to be close to 100% at the end of the cycle. The transmission rate value was also considerably lower than the prior value of 2.37 used in the model obtained from a study in Australia [22], and the value 1.78, obtained in a more recent study in the UK [21], implying that campylobacter spread through the flock at a slower rate than the flocks analysed in these studies.

Overall, our results differ from previous studies conducted in high-income countries, which have found that campylobacter spreads very rapidly among housed broiler chickens, and once the first bird becomes infected, the entire flock becomes infected in a matter of days [14–17]. To take into account the findings of these studies, and given the value of frequent sampling points for estimating the transmission rate of campylobacter within a farm, it was decided to increase the frequency of the sampling in the period of the production cycle where longitudinal data were supposed to be more informative (i.e. after day 10 to capture the early stages of the spread).

Our final dataset showed that the flock became infected during the early stages of the production cycle, with the first positive cloacal swabs and a boot sample being observed on day 15, and that the estimated prevalence at clearance was around 35%. These results are compatible with the low transmission rate obtained by the model, which was approximately 0.13 per bird per day (i.e. one infected bird could, on average, infect 0.13 birds per day). Absence of similar studies conducted in comparable settings prevented comparisons of our findings, so the reasons behind the differences from previous studies conducted in high-income countries can only be hypothesised.

One possible explanation for the slower transmission rate and final WFP is related to the environmental conditions in which the flock was raised. The longitudinal study was carried out between May and June 2017, when the weather in Irbid was very dry, and it has been reported before that survival of campylobacter is reduced in environments with low humidity [34, 35]. The density of birds within the studied flock could also have, to some extent, contributed for the lower transmission rate if compared with the studies previously mentioned. In the study by Van Gerwe *et al*. [22], conducted in Australian broiler flocks, broilers were kept under commercial/industrial conditions of 20 broilers per m^2^, whereas in our study, broilers were kept at a density of 10 broilers per m^2^, which is the typical semi-commercial practice in Jordan. Other variables compared with European or North American systems include the intensity of production, where management systems and dietary formulations may push broilers closer to their physiological limits.

At a bacterial level, different strains of campylobacter differ in their ability to infect chickens [11, 36], and different *C. jejuni* strains have distinct infection ecologies within the host [37]. In fact, it has been observed that a given strain of *C. jejuni* could inhibit another from invading the intestinal epithelium of the chicken, preventing colonisation [38]. Co-infection with multiple strains, presenting varied abilities to replicate and colonise chickens, may have affected the rate of transmission. Interestingly, Haag *et al*. [39] has also demonstrated that in mice, *C. jejuni* colonisation is dependent on the intestinal microbiota of the host. The intestinal microbiota composition and complexity of individual chickens is known to vary significantly within and between flocks [40, 41] and is likely to have been different here from flocks studied in other countries. The vaccination and pathogen exposure histories will also be highly variable, with notable examples including coccidia, Marek's disease, Gumboro disease and Newcastle disease. However, it should be noted that how the infection dynamics are affected by the characteristics of the pathogen, the co-infection with a mixture of strains and the microbiome composition are not fully known and yet to be explored in field conditions, even in high-income countries.

The flock was estimated to have become infected within the first 8 days of the production cycle. This was considerably earlier than comparable estimates from other studies. For example, in 40 Australian broiler flocks, all estimated times of first infection were >21 days [22]. A more recent study of broiler flocks in the UK estimated the time of first infection to be between 30 and 35 days for the majority of flocks [21]. The low biosecurity measures used in semi-commercial farms may have contributed to the persistence of campylobacter in the environment, explaining why it was detected earlier in the production cycle. On the other hand, the strict biosecurity measures maintained in high-income countries’ farms may have impeded campylobacter to persist in the environment from the previous production cycle, hence why it was detected later.

That campylobacter was first detected here 12 days after the initial infection is most likely explained by the infection transmission dynamics, being characterised by initially low prevalence that was likely missed by the sampling method (of 50 chicks per time point). Indeed, the prevalence remained very low until day 20, before increasing more rapidly and hence becoming more likely to be detected. The apparent lack of campylobacter infection prior to day 20 has been described in other studies [16, 22, 42] and it has been suggested that young chickens may be less susceptible to colonisation [24] because of anti-campylobacter maternally derived antibodies [26]. However, it is also likely that infections are routinely missed until the prevalence reaches more readily detectable levels. As stated above, early infection could be explained by the persistence of campylobacter in the environment caused by a lack of proper disinfection between broiler production cycles. Therefore, our study suggests that in this context, implementation of intervention strategies aimed at preventing campylobacter introduction and subsequent infection of the flock should be targeted at the first half of the production cycle, when infection is most likely to occur. The effect of interventions such as improved biosecurity measures has proved to be useful in decreasing broilers’ exposure to campylobacter in a previous study in the UK [43]. Implementing stringent biosecurity measures (like boot dips) has also been shown to have an effect on the prevalence of campylobacter, by reducing the transmission rate [20] and delaying the time when the flock first becomes infected [14, 44].

Further research is needed to evaluate the generalisability of our results to the epidemiology and transmission dynamics of campylobacter in other semi-commercial poultry flocks in Jordan, and other similar production systems in LMICs. However, from the results of the questionnaire, it is noteworthy that the expectation of experts on the prevalence of campylobacter in semi-commercial farms in Irbid were identical to the prevalence measured in our study at clearance (30%). This suggests, albeit anecdotally, that academics with expertise on campylobacter in Jordan have an accurate intuitive understanding of the prevalence of infection and thus that the degree of colonisation observed in our study may be quite typical of other semi-commercial farms, at least in Jordan.

## Conclusion

In this study, we estimated key epidemiological parameters driving the transmission dynamics of campylobacter in a semi-commercial poultry flock in Jordan using a method that could easily be adapted to other foodborne pathogens and livestock systems with similar dynamics. To the authors’ knowledge, this work represents the first longitudinal study estimating key within-flock transmission parameters for campylobacter infection in broiler chicken in the Middle East and LMICs in general. Our results suggest that the transmission dynamics of campylobacter in Jordan are significantly different from those observed in high-income countries, with an earlier introduction of the pathogen but a slower within-flock transmission. Future studies should focus on gathering data in other similar farms to determine the consistency and generalisability of the transmission dynamics of campylobacter. This would also allow exploration of how heterogeneities in conditions (e.g. environment, strain, temperature, density of birds) affect within-flock transmission dynamics in semi-commercial farms. Risk assessments for the latter steps of the food chain would also be important to provide an estimation of the risk of campylobacteriosis after consumption of chicken meat in Jordan, and to develop consistent and science-based principles to support food safety controls.
